# Supplementary material for: CREMSA: compressed indexing of (ultra) large multiple sequence alignments
Source: Bioinformatics. 2025 Jul 15;41(Suppl 1):i246–54. doi: 10.1093/bioinformatics/btaf211 (PMC12261481; doi:10.1093/bioinformatics/btaf211)
Supplement: btaf211_Supplementary_Data [file btaf211_supplementary_data.zip › btaf211_Supplementary_Data/Salson.272.marked.pdf]

Journal Title Here, 2024, 1–9
doi: DOI HERE
Advance Access Publication Date: Day Month Year
Paper

CREMSA: Compressed Indexing of (Ultra) Large Multiple Sequence Alignments

Mikaël Salson,[1,*] Arthur Boddaert,[2] Awa Bousso Gueye,[2] Laurent Bulteau,[3] Yohan Hernandez--Courbevoie,[2] Camille Marchet,[1] Nan Pan,[4] Sebastian Will[4] and Yann Ponty[4,*]

[1]Univ. Lille, CNRS, Centrale Lille, UMR 9189 CRIStAL, F-59000 Lille, France, [2]Département d'Informatique, Lille University, France, [3]CNRS UMR 8049 LIGM, Gustave Eiffel University, France and [4]CNRS UMR 7161 LIX, Ecole Polytechnique, Institut Polytechnique de Paris, France

*Corresponding authors. mikael.salson@univ-lille.fr; yann.ponty@lix.polytechnique.fr

FOR PUBLISHER ONLY Received on Date Month Year; revised on Date Month Year; accepted on Date Month Year

Abstract

Recent viral outbreaks motivate the systematic collection of pathogenic genomesin order to accelerate their study and monitor the apparition/spread of variants. Due to their limited length and temporal proximity of their sequencing, viral genomes are usually organized, and analyzed as oversized Multiple Sequence Alignments (MSAs). Such MSAs are largely ungapped, and mostly homogeneous on a column-wise level but not at a sequential level due to local variations, hindering the performances of sequential compression algorithms.

In order to enable an efficient handling of MSAs, including subsequent statistical analyses, we introduce CREMSA (Column-wise Run-length Encoding for Multiple Sequence Alignments), a new index that builds on sparse bitvector representations to compress an existing or streamed MSA, all the while allowing for an expressive set of accelerated requests to query the alignment without prior decompression.

Using CREMSA, a 65GB MSA consisting of 1.9M SARS-CoV 2 genomes could be compressed into 22MB using less than half a gigabyte of main memory, while executing access requests in the order of 100ns. Such a speed up enables a comprehensive analysis of covariation over this very large MSA. We further assess the impact of the sequence ordering on the compressibility of MSAs and propose a resorting strategy that, despite the proven NP-hardness of an optimal sort, induces greatly increased compression ratios at a marginal computational cost.

Key words: Indexing, Multiple sequence alignment, Compression

Introduction

The analysis of MSAs enables an evaluation of key metrics to understand molecular evolution, including conservation, coevolution, evolutionary distances, and other higher-order statistics. For instance, in viruses whose genetic material consists of single-stranded nucleic acids (ssRNA viruses), evolutionary constraints at the structural level can be revealed by covariation analysis. Such analyses motivate the analysis of the joint content of columns pairs, to assess the propensity of genomic positions to form a base pair mediated by hydrogen bonds. Ultimately, they enable a reconstruction of RNA architecture(s), potentially revealing targets for future drugs [Triebel et al., 2024].

Gene or genome-based alignments may feature extreme level of conservation at the column level, reflecting compact genomes undergoing pervasive evolutionary pressure. Such is the case of the genomic material of pathogens, collected upon an outbreak to monitor their evolution. This results in the presence of multiple near-identical sequences within alignments, featuring highly-homogeneous column contents. Compressing such alignments, especially at a column-wise level, can lead to spectacular compression ratios [Deorowicz et al., 2018]. However, such prior representations are mainly static: they require the MSA to be fully loaded in memory prior to their creation; they cannot be conveniently updated upon insertion of a new sequence; and most analyses will require a full uncompression of the alignment.

In this work, we introduce a new compressed index, called CREMSA (Column-wise Run-length Encoding for Multiple Sequence Alignments) which greatly reduces the storage required to store

© The Author 2024. Published by Oxford University Press. All rights reserved. For permissions, please e-mail: journals.permissions@oup.com

column-wise redundant MSAs. Contrasting with earlier efforts, solely focusing on the file-level compression of an MSA [Deorowicz et al., 2018], our index enabling direct and efficient access to column-wise statistics (no full decompression needed).

Our main contributions are the following:

- We introduce `CREMSA`, a novel compressed index for MSAs which supports optimized row and column-wise analyses;
- We investigate the impact of the genome order on the compressibility of MSAs. We formalize the problem and demonstrate its computational intractability
- We propose and benchmark several heuristic strategies for reordering MSAs. In particular, a lexicographic sort, based on a subset of lower-sequence identity columns, is seen to greatly optimize the compressibility of MSAs, even outperforming phylogenetic-based reordering. Moreover, this result does not depend on the compression algorithm being used;
- Beyond the reduced storage, `CREMSA` enables sigificantly faster access to column-wise statistics, enabling a covariation analysis of MSAs representing the evolution of large viral RNAs.

Materials and methods

Real-world and artificial datasets

Hunt et al. [2024] have re-processed millions of SARS-CoV-2 sequencing runs in order to provide accurate and reliable assemblies. They made 4.5 million SARS-CoV-2 assemblies available. From those sequences, we selected the most precise ones by removing all the sequences with at least 100 indeterminate nucleotides (any IUPAC nucleotide different from the canonical ones). We removed sequences featuring at least 2 consecutive `N`s, anywhere except at the sequence ends, in order to prevent excessive horizontal expansion (an unfiltered random subset of 10% sequences would result in a 230,481 columns MSA). In the end, we obtained 1,870,492 SARS-CoV-2 sequences that were aligned using Halign3 [Tang et al., 2022], the only multiple sequence aligner to run on a cluster node under 1.5TB RAM. The final alignment consists of 34,830 columns, totaling 65GB of data.

In order to further demonstrate `CREMSA`'s scope scope of validity, we considered an alignment of HIV-1 genomes, consisting of 5,381 sequences over 17,535 columns, retrieved from Los Alamos National Laboratory [2025] on March 2025. We also studied the compressibility of the Major Facilitator protein Superfamily (MFS) retrieved from Pfam [2025], which consists of 214,283 sequences aligned over 3,427 columns.

Finally, we generated artificial MSAs datasets, following 3 random models, in order to study the impact of the mutation rate, genomes ordering and varying parameters on the compression ratio. A model targets an expected pairwise *dissimilarity* δ, defined for a set of aligned sequences $\mathcal{G}$ (inc. gaps) as

$$\delta\left(\mathcal{G} := \{G\}_{i=0}^{S}\right) = \frac{1}{S(S-1)} \sum_{G_i \in \mathcal{G}} \sum_{G_{j>i} \in \mathcal{G}} d(G_i, G_j)$$

where $d(\cdot,\cdot)$ denotes the classic Hamming distance.

A first *independent model* starts from a poly-`A` sequence and, for each of the m targeted genomes and each of the n sites, performs a random mutation with probability $\delta/2$, resulting in an expected pairwise distance of δ as long as $\delta \ll 1/\sqrt{n}$. A second *phylogenetic model* simulates a basic evolutionary process driven by speciation events. Starting from a single ancestral poly-`A` genome G_0, $\mathcal{G} := \{G_0\}$, the process iteratively selects a genome $G \in \mathcal{G}$ uniformly, and replaces it with G' and G'', two modified versions of G respectively featuring $k-1$ and k mutations. The process stops when $\delta(\mathcal{G})$ exceeds its targeted value. A collection of m genomes, (sub)-sampled from $\mathcal{G}$ with replacement, is then organized in phylogenetic order (clade $\to$ compact intervals) and returned. It is worth noticing that such a (sub)sampling step typically, albeit imperfectly, preserves the expected pairwise dissimilarity. A final *shuffled model* returns a randomly-permuted set of sequences produced with the phylogenetic model.

Intuitively, the independent model represents a worst-case scenario for our compression philosophy, with column contents that are typically pairwise-inconsistent, offering limited opportunities for compression especially when a global order need to be preserved across columns. The phylogenetic model produces realistically divergent sequences, ordered in a manner that guarantees optimal compression ratios. Through an additional permutation (shuffled model) one tests the algorithm's capacity to correct inadequately-ordered sequences, in a context where a compression-friendly ordering is guaranteed to exist. For each model and for targeted dissimilarities in $[0.2‰, 0.5‰, 1‰, 5‰, 1\%, 5\%]$, we generated 10 MSAs consisting of 30,000 nucleotide sequences over 10,000 columns.

The `CREMSA` index

The central data-structure in our work is the *bit vector*: a $\{0,1\}$-array with fast *rank* and *select* queries. For a length-n bit vector B, $rank_1(B,i)$ is the number of 1s in the length-i prefix of B and $select_1(B,i)$ is the position of the i-th one in B, or $n+1$ whenever i is greater than the number of ones in B.

Index definition

Our compressed self-index, termed CREMSA, exploits the succession of identical letters (called *runs*) in the columns of a MSA, thus our approach is related to the table compression methods as reviewed by Giancarlo et al. [2009]. CREMSA indexes each column of the MSA independently to provide efficient column-wise queries. Each column C_j of length s of a multiple sequence alignment is stored using an approach close to run-length encoding by decomposing the column in two components: 1) a (compressed) bit vector B_j that identifies the start of each run in the column C_j and 2) a string that stores the repeated nucleotide of each run.

More formally, we define B_j of length s, as follows: $B_j[i] = 1$ iff $i = 1$ or $C_j[i] \neq C_j[i-1]$, for $1 \leq i \leq s$. The string N_j has a length of r_j, the number of runs in C_j, and is defined with: $N_j[i] = C_j[select_1(B_j, i)]$, for $1 \leq i \leq r_j$. An example is given in Figure 1.

Supported queries

Using *rank* and *select* queries on the bit vectors, the index supports the following basic queries:

- *access*(i,j) which returns the j-th nucleotide from the i-th sequence or, otherwise stated, the i-th nucleotide from C_j;
- *count-consecutive*(i,j) which counts the number of consecutive occurrences of $C_j[i]$ in C_j, *ie.* the length of the run of $C_j[i]$ in C_j at position i.

The *access*(i,j) query is solved using $N_j[rank_1(B_j,i)]$. The *count-consecutive*(i,j) query first identifies the first and final positions of the run, respectively b and e. The first position

C_1	C_2	C_3	C_4	C_5	C_6	C_7	C_8	C_9	C_{10}
C	G	C	A	C	A	A	A	C	C
C	C	C	A	C	A	C	A	C	C
C	C	C	A	C	A	A	A	C	C
C	-	C	A	C	A	A	A	C	C
C	-	C	A	C	A	C	A	C	C
C	C	C	A	G	A	C	A	C	C

B_1	B_2	B_3	B_4	B_5	B_6	B_7	B_8	B_9	B_{10}
1	1	1	1	1	1	1	1	1	1
0	1	0	0	0	0	1	0	0	0
0	0	0	0	0	0	1	0	0	0
0	1	0	0	0	0	0	0	0	0
0	0	0	0	0	0	1	0	0	0
0	1	0	0	1	0	0	0	0	0

N_1	N_2	N_3	N_4	N_5	N_6	N_7	N_8	N_9	N_{10}
C	G	C	A	C	A	A	A	C	C
	C			G		C			
	-					A			
	C					C			

Fig. 1: Left: a multiple sequence alignment of $s = 6$ sequences of length $n = 10$. Right: the representation of this MSA with the CREMSA index. The number of runs in each column C_j can be identified by the number of 1s in B_j. For instance, in column 2, the number of runs $r_2 = 4$. The strings N_j are represented vertically, *eg.* N_7 = ACAC. The last nucleotide in N_7 is a C because the last 1 in B_7 corresponds to the position in C_7 where a C is stored.

of the run is obtained with $b = select_1(B_j, rank_1(B_j, i))$ and the final position with $e = select_1(B_j, rank_1(B_j, i) + 1) - 1$, therefore, $count\text{-}consecutive(i, j) = select_1(B_j, rank_1(B_j, i) + 1) - select_1(B_j, rank_1(B_j, i))$.

Using those queries, one can devise more sophisticated queries in order to recover a complete sequence, or to compute the counts of each nucleotide in a column, the empirical entropy of a column, the empirical entropy of a pair of columns, the mutual information of a pair of columns, the G-test as promoted by R-scape [Rivas et al., 2017], the conservation score of RNAalifold [Bernhart et al., 2008], etc.

Counting nucleotides. The counts of each nucleotide in a column j are obtained by iterating on each 1 in B_j (using $select_1$) and by computing *count-consecutive* for the position of each 1 as shown in Algorithm 1. Once the counts have been obtained, computing the empirical entropy and other site-level metrics is straightforward.

Algorithm 1 Counting occurrences of all nucleotides/gaps in column j

```
function COUNT(j)
    occurrences = {}
    s ← |B_j|
    i ← 1
    b ← 1
    while i ≤ s do
        m ← count-consecutive(i, j)
        Increment occurrences[access(i, j)] by m
        i ← select_1(B_j, b + 1)
        b ← b + 1
    end while
    return occurrences
end function
```

Those queries can easily be generalized to more than one column in order to study the covariations between columns. For instance, $count\text{-}consecutive(i, j_1, j_2)$ would return the length of the run of $C_{j_1}[i] \cdot C_{j_2}[i]$ in the pair of columns C_{j_1}, C_{j_2} at position i. This can be achieved by identifying the start position b of the run at position i in B_{j_1} and B_{j_2}, and by keeping the highest one. Conversely, for the end position e, we keep the lowest one among the ones in B_{j_1} and in B_{j_2}. We can compute this with $b = \max(select_1(B_{j_1}, rank_1(B_{j_1}, i)), select_1(B_{j_2}, rank_1(B_{j_2}, i)))$ and $e = \min(select_1(B_{j_1}, rank_1(B_{j_1}, i)), select_1(B_{j_2}, rank_1(B_{j_2}, i)))$. Then, $count\text{-}consecutive(i, j_1, j_2) = e - b + 1$. We can generalize the approach for $count\text{-}consecutive(i, j_1, j_2, \ldots, j_k)$. Using those counts, one can then compute the empirical entropy for a pair of columns, or for k columns.

Index construction

To construct CREMSA, from a multiple sequence alignment with s rows and n columns, a naive solution consists in initializing n length-n bit vectors. Then by comparing each sequence in the MSA to the next one, it is straightforward to identify the start of the runs in each bit vector.

However, from a practical point of view, this requires to store $n \times s$ bits in memory during the construction, before the bit vectors are actually compressed, which would be several orders of magnitude larger than the final index. One solution would be to use dynamic compressed bit vectors [Prezza, 2017]. They would always be compressed, preventing the issue of storing $n \times s$ plain bits, while allowing one to update them to set bits at 1 during the construction. This advantage however comes with a logarithmic penalty at query time. Another solution is to split the MSA into manageable bundles of genomes (entire rows), create a CREMSA compressed index for each bundle and then merge them progressively. Once a bundle is ready, it is merged to the existing compressed index.

The merge between two CREMSA is performed column by column. Ensuring that the last row of the first index and the first row of the second index represent the same genome simplifies the process. A new bit vector is created from the concatenation of the bit vectors of both index, excluding the first bit of the second index. The same occurs for the N_j strings. As the merge replaces the second index, the new bit vector and N_j string replaces the pre-existing ones in the second index.

Complexities

When constructing CREMSA, let S be the number of sequences stored in a bundle. The approach needs to store $n \times S$ plain bits, plus the compressed index so far. Therefore the additional space used compared to the final CREMSA is $n \times S$ bits. At step i, we need to merge an index of S sequences into an index of $(i-1)S$ sequences, representing $(i-1)nS$ bits in compressed form. As we need to merge $\frac{s}{S}$ bundles, the time complexity is $\sum_{i=1}^{\frac{s}{S}}(i-1)nS = \Theta(\frac{ns^2}{S})$. With $S = \frac{s}{\log s}$, this leads to $o(ns)$ extra space and $\Theta(ns \log s)$ time.

As the *rank* and *select* operations can be computed in constant time on compressed bit vectors, as reviewed by Navarro and Mäkinen [2007], the *access* and *count-consecutive* queries are performed in constant time. Any genome of length n can thus be retrieved in $\Theta(n)$ time, making CREMSA a self-index as it can recover the input data. Counting the occurrences of each nucleotide or computing the empirical entropy of a column j is obtained in $\Theta(r_j)$ as the while loop in Algorithm 1 iterates on each 1 in B_j. Counting pair of nucleotides in pair of columns j_1, j_2 follows the same principle, hence the time complexity in $\Theta(\max(r_{j_1}, r_{j_2}))$.

Since our genomes are highly redundant, the bit vectors B_j are expected to be very sparse. In such cases, the compressed bit vectors only consist in storing the relative positions of the 1s in each bit vector, which leads to a $\Theta(r_j)$ space complexity. When B_j is dense, the space complexity is $O(H_0(B_j))$, where H_0 is the zero-th order empirical entropy of B_j.

Both our time and space complexities depend on r_j the number of runs in column j, meaning that decreasing this value makes the index more time and space efficient.

Improving compressibility through sequence reordering

The number of runs in a column of the MSA critically and directly depends on how the genomes are ordered in the MSA. Ideally, we would like to find an ordering which maximizes the average lengths of runs across columns, *i.e.* minimizes the total numbers of runs.

Unfortunately, finding an ordering of the sequences minimizing the total number of runs is NP-complete, as can be seen with the following simple reduction from Hamiltonian Path. Given a graph $G = (V, E)$ where all vertices have degree 3, build $|V|$ length-$|E|$ sequences over alphabet $\{\mathtt{A}, \mathtt{U}\}$, such that the jth character of sequence i is U iff edge j is incident to vertex i. Then each column has 5 runs in the worst case: two length-1 runs of U separating three runs of A. The number of runs decreases by 1 or 2 if the column starts and/or ends with U; independently of the order of the rows, this amounts to exactly 6 missing runs in total since each vertex has degree 3. Also, the number of runs in column j decreases by 2 if both occurrences of U are consecutive, in which case we say that edge j is *realized*. The overall number of runs is thus $5|E|-6-2\times$number of realized edges. Each pair of consecutive rows can realize at most one edge (if the corresponding vertices are adjacent in G), so the number of runs is at least $5|E|-6-2(|V|-1)$, and this bound is attained if and only if the ordering of the vertices chosen for each row forms an hamiltonian path in G.

Even though finding an optimal order is computationally intractable, simple heuristics may still help find better orders than a random one. Břinda et al. [2024] addressed a similar problem: they used phylogenetic information in order to improve compression ratios of microbial genomic sequences. In particular, they reordered 590,779 SARS-CoV-2 genomes using GISAID phylogeny. They achieved a 1,647 compression ratio sorting sequences by their phylogenetic order and compressing them with `xz`. The same order can also benefit `CREMSA`: clustering the most similar sequences together should produce longer runs. We evaluate two levels of phylogenetic information for SARS-CoV-2: a coarse-grained information, the Pango nomenclature [Rambaut et al., 2020], which is a manual curation of clusters in the phylogenetic tree; and a fine-grained information based on the whole phylogenetic tree.

We also introduce a new ordering, specific to multiple sequence alignments that does not depend on an external (or heavy to compute) information, such as the phylogeny of the sequences. Let $G_1, \ldots, G_s$ be the s genomes in the MSA. Let occ_j be an array of the number of occurrences of each character in column C_j, then the fraction of identity id_j in C_j is $id_j = \frac{\max(occ_j)}{s}$. Using the COUNT function on `CREMSA`, one can quickly compute the fraction of identity in C_j. Thus, we will extract the d positions $p_1, \ldots, p_d$ of the columns having the lowest fraction of identity, with $id_{p_1} < id_{p_2} < \cdots < id_{p_d}$. Then, for each genome G_i, we extract a word w_i of d characters, such that $w_i = G_i[p_1] \cdot G_i[p_2] \cdots G_i[p_d]$, this is a shuffled subword of G_i. We call those d-length shuffled subwords d-discriminative subwords. The genomes $G_1, \ldots, G_s$ are eventually sorted according to the lexicographic order of the d-discriminative subwords. Once the positions $p_1, \ldots, p_d$ are identified, the d-discriminative subwords can be sorted in linear time $\Theta(sd)$ and space $\Theta(s)$ using a bucket sort. This whole process can be directly performed on a `CREMSA` index without the input data. Once the new order is found, each column is successively decompressed, updated to reflect the new order, and recompressed.

Entropy and covariation metrics for RNA comparative analysis

Columnwise entropy is a common metric of the local diversity of MSA. It is defined for a column j as $H_j = \sum_x p_x^j \log(p_x^j)$, where p_x^j denotes the frequency of character x in the j-th column.

Various covariation measures have been discussed in the literature; among them several variations of the general theme of mutual information [Gutell et al., 1994]. In this work, we provide data for two of these scores. First, we study the G-test [Woolf, 1957], which was found to have best performance in `R-scape` [Rivas et al., 2017]. in their comparison of eight different covariation scores. The G-test score $\mathrm{GT}(j, j')$ for two alignment columns j and j' is defined as

$$GT(j,j') = 2\sum_{x,y} N_{jj'} p_{x,y}^{j,j'} \log \frac{p_{x,y}^{j,j'}}{p_x^j p_y^{j'}},$$

where for nucleotides x and y, as well as columns j and j', $N_{j,j'}$, $p_{x,y}^{j,j'}$, p_x^j, $p_y^{j'}$ respectively denote the number of base pairs; the probability of the nucleotide pair x,y; the probability of x in j and the probability of y in j'. In this score we consider only determined nucleotides A, C, T/U, or G and their pairings, but consider all kinds of canonical pairings, i.e. AU, GC, or GU, and non-canonical pairings.

Second, we consider the RNAAlifold score as introduced by Bernhart et al. [2008] to predict canonical RNA secondary structures based on MSAs. This score distinguishes between canonical pairing and non-canonical pairing, which is penalized as incompatibility. The score is defined as sum of two components, a covariation score $g_{\mathrm{cov}}(j, j')$ and an incompatibility score $g_{\mathrm{inc}}(j, j')$. The covariation score is a sum-of-pairs score over the Hamming distance of canonical base pairs occurring in columns j and j' in all pairs of alignment rows. The incompatibility score penalizes gap symbols in columns j and j' as well as non-canonical base pairs.

Results

`CREMSA` is implemented in C++ using the sdsl-lite v3 library[1] and is available at `https://gitlab.univ-lille.fr/cremsa/cremsa`, or as a Docker image at `https://hub.docker.com/repository/docker/mikaels/cremsa/` under a GNU GPL v3 license. `CREMSA` uses two types of bit vectors in sdsl-lite depending on the number of runs in the bit vector: either SD bit vector or RRR bit vector. A SD bit vector is intended to support sparse bit vectors, their compression scheme relies on gap-encoding. Thus we use them in `CREMSA` for any bit vector B_j with $r_j/s < .1$. In the other cases, a bit vector using the RRR compression method [Raman et al., 2007] is used because it is less space consuming with denser bit vectors. In practice, the *rank* operation is in $O(\log(\frac{s}{r_j}))$ in the SD vector

[1] `https://github.com/xxsds/sdsl-lite`

	Compression ratio	Runtime (s)	Memory (MB)
gzip	3	12,658	2
xz	2,087	9,136	97
CoMSA	5,285	3,618	66,429
CREMSA	2,922	413	529
CREMSA *	2,922	859	641

Table 1. Compression ratios of various compressors on the reordered multiple sequence alignment of 1,870,492 SARS-CoV-2 genomes (65GB uncompressed). The higher the compression ratio, the better the compression. CREMSA* includes the time for reordering the sequences, directly using the CREMSA index. gzip and xz were launched with the default option and with the best compression level (-9). The results with the latter were similar or even worse, thus only the default option is shown.

Order	gzip	xz	CoMSA	CREMSA
Initial	3	1,211	4,628	965
Length	3	1,265	4,770	1,135
Pango	3	1,537	4,891	1,316
Pango+length	3	957	4,962	1,741
Phylogenetic	3	2,391	5,322	1,895
Random sample	3	1,006±5	5,035±12	1,204±103
Discriminative	3	2,087	5,285	2,922
Discriminative+pango	3	2,087	5,285	2,925

Table 2. Compression ratios on the multiple sequence alignment of 1,870,492 SARS-CoV-2 genomes (65GB uncompressed) reordered in various ways. The higher the compression ratio, the better the compression. The order are the following ones. Length: by genome length, pango: by pango lineage, pango+length: by pango lineage and genome length, phylogenetic: pre-order traversal of the phylogenetic tree, random sample: by 5,000 random subwords (the process was repeated 50 times, the average and standard deviation are shown), discriminative: by the 5,000-discriminative shuffled subwords, discriminative+pango: by the latter and by the pango lineage.

and $O(1)$ for the RRR vector. The *select* operation is in $O(1)$ for SD vectors and $O(\log s)$ for RRR vector. CREMSA also includes the reordering of the sequences based on their d-discriminative subwords, either at construction or afterwards.

We built CREMSA on the 1,870,492 SARS-CoV-2 genomes. When storing the CREMSA index on disk, the compressed bit vectors as well as the nucleotides stored in each N_j string are compressed using gzip. When performing the queries, the whole index is loaded in memory and both the bit vectors and the N_j strings are un-gzipped. Note that the bit vectors are still compressed by the scheme used in the SDSL library. As far as we know, there exists no other index structure for multiple alignments. However we compare our compression results to other compressors.

All experiments were performed on the single thread of a computer with a 16-core Intel Xeon Gold 6130, 128GB of memory and a 50TB NFS-mounted hard drive . CoMSA ran on several threads as there is no option to force it run on a single thread. The times shown include user and system times.

Compression ratios

CREMSA stores a multiple sequence alignment in a compressed representation that also allows efficient queries. However CREMSA does not store the identifiers of the sequences it represents. For the sake of comparison, the compressors were assessed on the basis of the sequences only, the sequence identifiers were not taken into account. Table 1 shows the compression ratios on the SARS-CoV-2 dataset of CREMSA, of two generic compressors (gzip and xz) and one specialized compressor dedicated to multiple sequence alignments (CoMSA [Deorowicz et al., 2018]) on the 1.9M genomes reordered according to our heuristic based on 5,000-discriminative shuffled subwords, which appeared to be the best order for this dataset (see Supp. Fig. 1).

Unsurprisingly, CoMSA is the best compressor, however, this comes at the cost of prohibitive memory usage. CREMSA is very competitive in terms of compression ratio compared to xz. In the reordered version, CREMSA even compresses better than xz while being an order of magnitude faster. gzip performances are poor because of its look-back buffer whose size is only 32,768 characters long, slightly too small to contain more than a single genome (the aligned genomes are 34,830 characters long).

As introduced previously, the order of the sequences matters for CREMSA in order to minimize the number of runs in each column. Reordering the sequences also benefits dictionary-based compressors since similar sequences are closer. We assessed the impact of the following orders on the compression:

1. by increasing length of the genomes;
2. by pango lineage (*ie.* the lexicographical order of their pango lineage);
3. by pango lineage and length of the genomes;
4. by a pre-order traversal of the phylogenetic tree;
5. by the shuffled 5,000-discriminative subwords
6. by 5,000-random subwords (same idea as before but the 5000 columns are chosen randomly instead of using the fraction of identity)

In Table 2 we show how the order influences the size of the compressed representation of the sequences in the MSA.

The phylogenetic order improves compression in all the cases, confirming the result by Břinda et al. [2024]. Adding information about the length of the sequences improves the compression for CREMSA but decreases it for xz. This is probably due to the nature of the compression methods. While CREMSA compresses column-wise, where it is an advantage to have gaps at the ends clustered together, xz compresses sequence-wise, where there is no such advantage. Finally, the discriminative order allows the best compression ratios for all the methods (except gzip that cannot benefit from the redundancy for reasons explained previously). Under that order, adding phylogenetic information does not bring any improvement. Strikingly, the discriminative order even outperforms the full phylogenetic order on CREMSA.

The performance of the discriminative subwords increases with longer subwords and then plateau at 5,000 (see Supp. Fig. 1). Surprisingly the performances of the discriminative order is not negatively impacted when shuffling the discriminative subwords used, and can even improve compressibility, as witnessed for values of d between 500 and 5,000 on the 1.9M SARS-CoV-2 dataset.

We report in Supp. Tables 1 and 2 the compression ratios obtained by CREMSA on the HIV and MFS datasets. We observe typical compression ratios of ~20x for both the HIV and MFS datasets. These compression ratios are much more modest than those observed for the SARS-CoV-2 dataset (~3000x), but ultimately reflect a significant decrease in average sequence identity (92.6%/91.4% for HIV/MFS, versus 99.7% for SARS-CoV-2). The compression ratios observed on these, substantially more diverse, alignment are much lower since those datasets

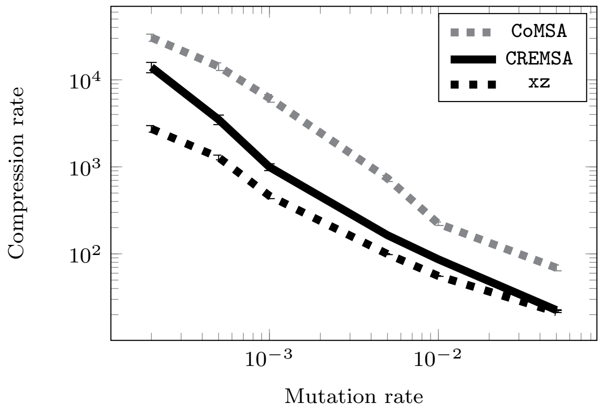

Fig. 2: Compression rate of CoMSA, CREMSA and xz on the shuffled model for the artificial dataset for .02% to 5% mutation rates. The compression rate is computed after removing the constant space taken by each compressor.

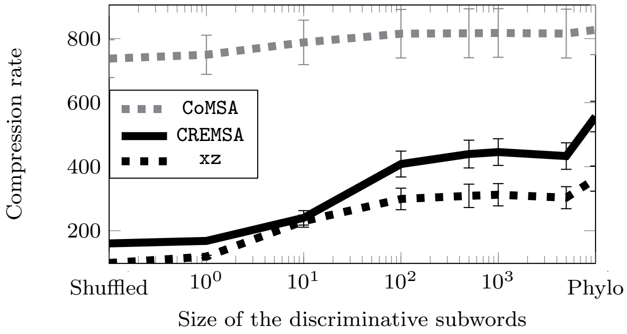

Fig. 3: Compression rates of CoMSA, CREMSA and xz on the artificial dataset with .5% mutation rate, depending on the sequence ordering. The runtime is the user+system time. The memory is the peak RAM usage at construction. Shuffled and Phylo refer to the corresponding model in the artificial dataset. All the values in-between correspond to the shuffled models reordered according to our discriminative shuffled subwords, of different lengths.

are much less redundant. For the same reason, the order only marginally impacts the compression ratios. However, CREMSA globally has a compression ratio similar to xz despite the lower redundancy and the larger alphabet (for MFS). This result is consistent with our artificial dataset (Fig 2) where CREMSA is consistently better or similar than xz and where the compression rate quickly drops with larger variability.

As already shown on the SARS-CoV-2 dataset, the sequence order matters for the compression rate, especially for CREMSA. On our artificial datasets, we confirm that the compression rate of CREMSA and even on xz or CoMSA can be significantly improved through our discriminative ordering (Fig. 3, Supp. Fig. 3 for a highly mutated dataset, and Supp. Fig. 2 for the huge effect it has on CoMSA on lowly mutated dataset).

Runtime analysis

Construction time. Constructing a CREMSA index offers a space-time trade-off. By default, CREMSA reads chunks of 100,000 sequences

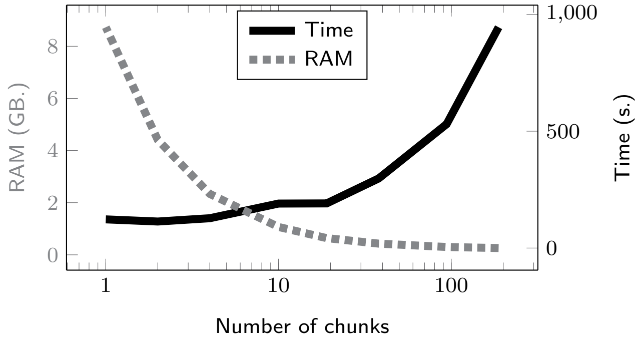

Fig. 4: Time and maximal RAM consumption, observed for different number of chunks, for the online index construction on 1,870,492 SARS-CoV-2 genomes.

Nb seq.	10k	50k	100k	250k	500k	1M	1.5M	1.87M
Time (ns)	28	37	44	55	67	89	103	111

Table 3. Average time per nucleotide to perform the *access* query on an index of 10,000 to 1.9M SARS-CoV-2 genomes.

and stores an uncompressed version of the bit vectors, representing $100,000n$ bits in memory. The chunks are then progressively merged in the compressed index. Once all the sequences have been processed, the index is gzipped and written on disk. The time and memory consumption of CREMSA construction on 1.9M genomes is shown in Table 1. Obviously, loading larger chunks reduces the construction time and increases the memory consumption (Fig. 4). As expected from the construction complexities, a logarithmic number of chunks offers the best trade-off.

Access to a random nucleotide. The purpose of CREMSA is not only to compress a multiple sequence alignment but also to provide efficient queries on it. For instance, CREMSA provides random access to any nucleotide in the multiple sequence alignment, something that competing compressors cannot achieve without first decompressing the whole content of a column (or alignment).

We assessed the *access* query by retrieving 10,000 sequences uniformly chosen within various CREMSA indices ranging from 10,000 to 1.9M sequences. The experiments were repeated 10 times. The mean time for the *access* query is shown for each experiment in Table. 3.

Our experiments show that the *access* query is not performed in constant time. This is due to the implementation of the sparse bit vector we chose, which doesn't have a constant time complexity for *rank* queries, as explained previously. However, when the number of sequences increases ~190 fold, the access time increases less than 4 fold, showing that the *access* query scales very well, at about 100ns for an index of 1.9M genomes.

Impact of the number of runs per column. The time complexities directly depend on the number of runs in each column of the MSA. As expected, from the compression ratios, the number of runs is lower in the discriminative order compared to the initial order. More precisely, there are more than .1% of runs (*ie.* more than 1,870) in less than 5% of the columns in the discriminative order, and in 12% of the columns in the initial order (see Supplementary Figure 6). The median number of runs is 95 (0.005%) with the discriminative order and 141 (0.008%) in the initial order.

Counting nucleotides in a column. The runtime of queries on the columns depend on their number of runs. We compared the runtime for the COUNT function in columns with 1 to almost 10,000 runs. For each $i \in \{1, \dots, 4\}$, 500 columns were randomly chosen with a number of runs between $0.5 \cdot 10^i$ and $2 \cdot 10^i$. For each selected columns, the COUNT function is run 10 times. The results are shown in Figure 5.

In theory, the COUNT query is linear in the number of runs ($\Theta(r_j)$). In practice, other considerations come into play such as cache efficiency making COUNT more efficient on columns with fewer runs than those with more runs.

Queries on multiple columns. It could be argued that an index is not required to compute counts on each column, since such statistics could be precomputed in a single run, and subsequently queried directly. However, this is not the case anymore when one wants to query counts on multiple columns. For instance, for pair count queries, each column is queried on average $O(s)$ times.

Optimizing genome order is all the more important for pair count queries since the time complexity depends on the largest number of runs among the two columns. In practice, the optimized order is 4 times quicker to compute pair count queries than the initial order on the 1.9M genomes: the counts for the 607M pair of columns are computed in 5h and 81MB of RAM under the 5000-discordant subword order and in 22h and 197MB of RAM under the initial order. Interestingly, the phylogenetic order is almost as slow as the initial order for this pair count query.

Covariation analysis of SARS CoV-2 genomes

In order to demonstrate the usability of our index in the context of very large viral alignments, we analyzed a 65GB large MSA consisting of 1,870,492 SARS-CoV2 genomes, further described in the Datasets subsection. More specifically, we strived to identify sites and regions undergoing evolutionary pressure.

More specifically, we analyzed the columnwise entropy as a measure of the diversity of nucleotides observed within a given column. Pair statistics were also considered to capture a notion of coevolution, and we considered the `GTest` statistics used at the core of the popular `RScape` method [Rivas et al., 2017]. This score is closely related to Mutual Information, and does not specifically focus on the propensity of sites to form a base pair, rather rewarding an observed bias of evolution away from the uniform distribution. Towards structural analysis, it is sometimes beneficial to complement such metrics with scores that explicitly reward compatible nucleotide pairs, more likely to indicate compensatory mutations. We thus turn to a conservation score introduced by `RNAalifold` [Bernhart et al., 2008] as a pseudoenergy term to complement the classic Turner energy model with evolutionary information.

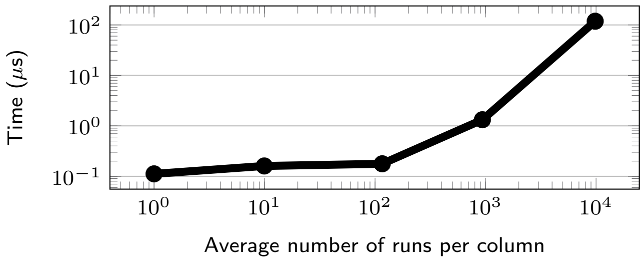

Fig. 5: Average time to count occurrences of nucleotides in a single column of 1.9M genomes, depending on the number of runs.

We compute the site-level entropy, for each of the columns in the alignment, and the two-sites `GTest` and `RNAalifold` scores for each pair of columns. As mentioned in the Runtime analysis Section, an optimized ordering of genomes allows performing the whole computation in little less than 5h on a single CPU, an impressive feat given the large number of column pairs (606,547,035), each requiring an iteration over 1,870,492 rows in an uncompressed setting.

For the sake of readability, and given the length of the alignment, we present in Figure 6 a coarse-grained visualization of the results. Namely, the alignment is broken up into 1,000nts non-overlapping regions, and we report the number of sites and pairs having value greater than a metrics-specific cutoff (entropy > 0.1, `GTest` > 250k, `RNAAlifold` > 0.75).

On a single-site level, our analysis shows that only a marginal fraction (1-30‰) of the positions feature some level of entropy (above 0.1). Such a perceived conservation likely reflects strong evolutionary constraints, coupled with a close temporal proximity of collection for the majority of genomes, in the initial stages of the COVID 19 outbreak. Interestingly, the largest values observed for the columnwise entropy are found in the 22k-26k region of the alignment, encoding the *spike* glycoprotein. Since this protein has been targeted by vaccines, higher mutation rates in this genomic region are expected and consistent with documented escape strategies (along with the position of variants of concern). Probably more surprising is the presence of a steady, relatively high, level of mutations in the terminal 25k-35k region, despite the presence in this region of multiple, sometimes overlapping, open reading frames.

Our two chosen metrics are in general agreement in the region-level coevolutionary analyses, but also feature notable discrepancies. On a broad level, our analyses reveal the existence of a hub in the 24k-25k region of the MSA, associated with the ORF encoding the spike protein. According to both metrics, this region seemingly coevolves with 10 out of 23 regions in 5', and with all of the terminal regions (25k-35k). Both metrics also support a strong and pervasive coevolution with the 25k-35k region, both on an information-theoretic and structural level. As expected, the `RNAalifold` score appears more selective than the `GTest`, leaving some regions entirely devoid of coevolving partners, while highlighting a potential of the 4k-5k region to region with downstream regions which are not visible from the `GTest`. This is likely due to the *transitive* nature of scores akin to a Mutual Information (if both pairs (a, b) and (a, c) have large MI, then (b, c) typically has large MI). Conversely, the large number of interactions having good `RNAalifold` score between the 21k-22k and 24k-25k regions, not present in the `GTest` data, suggests the presence of compensatory mutations, possibly indicating evolutionarily-conserved structural elements.

While those promising preliminary results would certainly warrant further refinements (*e.g.* phylogenetic subsampling, comparison against available probing data [Manfredonia et al., 2020]), we must stress that the mere production of the metrics would not have been realistically feasible without an index as efficient as `CREMSA`, thus demonstrating its value and relevance.

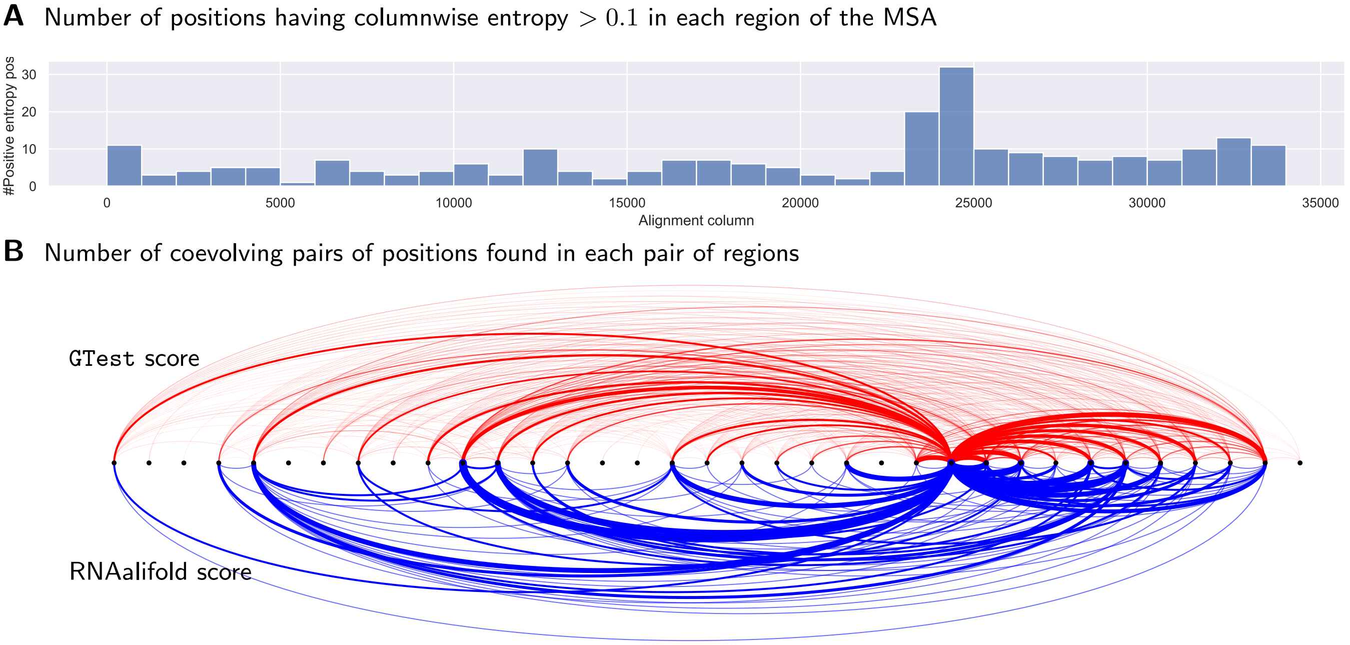

Fig. 6: Main coevolving regions and entropic positions derived from an ultra-large MSA consisting of 1,870,492 SARS-CoV 2 genomes. The top panel (A) represents as a barplot the number of positions, in each region (1k nts slice) of the genome, associated with a columnwise entropy greater than 0.1. The bottom panel (B) shows main coevolving regions, each dot representing 1,000 consecutive nucleotides, with respect to two classic metrics (top–`GTest`, bottom–`RNAalifold` conservation score). For a given metrics and pair of region, the strength of the interaction (line thickness) indicates the number of column pairs which exceed a predefined cutoff.

Discussion

We have introduced a new compressed index for highly redundant multiple sequence alignments, as well as a new ordering of mutiple sequence alignment that benefits other compressors. Our index efficiently compresses the input data using compressed bit vectors. `CREMSA` is superior to `xz` in terms of compression ratio on almost all the cases considered. Surprisingly we noticed on most DNA samples that `xz -9` obtained poorer compression ratios compared to `xz -6` (see Supp. Fig 5). `CoMSA` uses a more efficient compression scheme, but more resource demanding, based on the positional Burrows-Wheeler transform (pBWT). The pBWT was initially introduced to index haplotypes represented as bit vectors [Durbin, 2014]. As the pBWT, and other BWT-based indexes, can efficiently query the indexed text, one may wonder whether we could have the best of both world: compressing as efficiently as `CoMSA` while providing the efficient queries `CREMSA` allows. However BWT-based indexes can query patterns or maximal exact matches, which are not the queries of interest in our case. On the contrary, we want to efficiently enumerate all the possible tuples of letters in any tuple of columns. As far as we know, there is no direct method to get counts over unrelated columns/sequences in a pBWT or in any other BWT-related index. Thus, the query we are interested in within this paper could only be obtained with a BWT-based index by decompressing the corresponding columns of the MSA, leading to a linear-time algorithm, while `CREMSA` is linear in the number of runs.

The new order we introduce significantly improves compression. On the artificial datasets, it is clear that our ordering brings the compression ratio much closer to what the phylogenetic order achieves. It is noteworthy that on the SARS-CoV-2 dataset our order for `CREMSA` even outperforms the phylogenetic order. Our hypothesis is that technical artifacts (such as sequence length, sequence errors, undetermined nucleotides…) are well captured by the discriminative subwords while they are not in a phylogenetic order. The size of the word to use (d) for the reordering should be larger for more similar datasets. On the artificial datasets, the best value seeemed to be around $d = 100$ for low mutations (Fig 3) and can be as low as $d = 10$ for larger mutation rates (Supp. Fig. 3). It is yet to be determined whether other simple orders could go beyond the compression ratios we achieved. It is surprising that the order of the letters within the subword has no significant impact on the compression ratios. This suggests that some information used for the ordering is redundant and that a more careful selection of the columns could achieve similar results.

The wealth of data used for this kind of analysis also comes with its own limitations. Among millions of genomes, some of them will be of poor qualities, despite the filters we have applied. Those genomes will tend to fragment the MSA by introducing gaps. They could prevent the identification of compensatory mutations, when those mutations end up being spread on different columns because of gaps. Among millions of genomes, it is usually difficult to identify the tiny fraction of them which is problematic. Using `CREMSA` one could easily identify the genomes which often introduce gaps in the alignment. Thus, `CREMSA` could help improve the public datasets of millions of genomes and it could eventually help to identify meaningful covariations.

`CREMSA` could easily be turned into a fully dynamic structure, allowing the insertion of new sequences to an existing index *on the fly*. However in such a case, the computation of the multiple sequence alignment would remain the bottleneck, as

the MSA should be recomputed. As mentioned previously the computation of the MSA on 1.9M SARS-CoV-2 genomes required 1.2TB of RAM with Halign3. This makes such computation not readily accessible. Thus the availability of `CREMSA` motivates the development of more frugal approaches to multiple sequence alignment, tailored for highly similar sequences.

Conclusion

`CREMSA` is a frugal compressed index for highly redundant multiple sequence alignments achieving compression ratios close to a state-of-the-art specific compressor, but taking a fraction of the resources. Additionally, `CREMSA` provides random access to any nucleotide and greatly eases the computation of multi-columns/sites statistics in the MSA, to determine valuable covariation statistics.

We also introduced a new ordering on multiple sequence alignments that also benefits other compressors. Such an optimized ordering is purely beneficial for `CREMSA` (as well as other compressors), as they both reduce space and time consumption of our structure, and enable faster queries. Namely, in our running example focusing on SARS-CoV 2, our new order reduces the index size by a factor of three and the computation time for pair count queries by a factor of four.

We also compute an index on 1,870,492 SARS-CoV-2 genomes aligned with Halign3 (65GB of data) in an index compressed to 22MB on disk and taking 76MB of main memory. Then, in a few hours `CREMSA` could compute pair counts for any pair of columns, that could then be used to derive conservation scores.

Data availability and Reproducibility

The multiple sequence alignment of SARS-CoV-2 genomes is available on Zenodo at `https://zenodo.org/records/14698859`, the other datasets (HIV, MFS and artificial datasets) are available at `https://zenodo.org/records/15100011`. A Snakefile is available to reproduce the benchmarks on `CREMSA` and the other compressors at `https://gitlab.univ-lille.fr/cremsa/bench`.

Acknowledgment

This work was funded by the French *Agence Nationale de la Recherche* (ANR) through the INSSANE project (ANR21-CE45-0034). Additional computing resources where provided by the *Institut Français de Bioinformatique* (IFB) and the IFB Core cluster, under the ANR-funded *Programme d'Investissements d'Avenir* (RENABI-IFB; ANR-11-INBS-0013 and MUDIS4LS ANR-21-ESRE-0048).

We are also grateful to Karel Břinda for pointing us to the Hunt et al. dataset.

References

S. H. Bernhart, I. L. Hofacker, S. Will, A. R. Gruber, and P. F. Stadler. RNAalifold: improved consensus structure prediction for RNA alignments. *BMC Bioinformatics*, 9(1), Nov. 2008. doi: 10.1186/1471-2105-9-474.

K. Břinda, L. Lima, S. Pignotti, N. Quinones-Olvera, K. Salikhov, R. Chikhi, G. Kucherov, Z. Iqbal, and M. Baym. Efficient and robust search of microbial genomes via phylogenetic compression. *bioRxiv*, 2024. doi: 10.1101/2023.04.15.536996.

S. Deorowicz, J. Walczyszyn, and A. Debudaj-Grabysz. CoMSA: compression of protein multiple sequence alignment files. *Bioinformatics*, 35(2):227–234, 07 2018. doi: 10.1093/bioinformatics/bty619.

R. Durbin. Efficient haplotype matching and storage using the positional burrows–wheeler transform (pbwt). *Bioinformatics*, 30(9):1266–1272, 2014.

R. Giancarlo, D. Scaturro, and F. Utro. Textual data compression in computational biology: a synopsis. *Bioinformatics*, 25(13):1575–1586, 2009.

R. R. Gutell, N. Larsen, and C. R. Woese. Lessons from an evolving rRNA: 16S and 23S rRNA structures from a comparative perspective. *Microbiol. Rev.*, Mar. 1994. doi: 10.1128/mr.58.1.10-26.1994.

M. Hunt, A. S. Hinrichs, D. Anderson, L. Karim, B. L. Dearlove, J. Knaggs, et al. Addressing pandemic-wide systematic errors in the sars-cov-2 phylogeny. *bioRxiv*, 2024. doi: 10.1101/2024.04.29.591666.

Los Alamos National Laboratory. Los alamos hiv sequence database, 2025. `https://www.hiv.lanl.gov/`.

I. Manfredonia, C. Nithin, A. Ponce-Salvatierra, P. Ghosh, T. K. Wirecki, T. Marinus, N. S. Ogando, E. Snijder, M. J. van Hemert, J. M. Bujnicki, and D. Incarnato. Genome-wide mapping of sars-cov-2 rna structures identifies therapeutically-relevant elements. *Nucleic Acids Research*, 48(22):12436–12452, 11 2020. doi: 10.1093/nar/gkaa1053.

G. Navarro and V. Mäkinen. Compressed full-text indexes. *ACM Computing Surveys (CSUR)*, 39(1):2–es, 2007.

Pfam. Pfam models and alignments on interpro, 2025. `https://www.hiv.lanl.gov/`.

N. Prezza. A framework of dynamic data structures for string processing. *arXiv*, 2017. doi: 10.48550/arXiv.1701.07238.

R. Raman, V. Raman, and S. R. Satti. Succinct indexable dictionaries with applications to encoding k-ary trees, prefix sums and multisets. *ACM Transactions on Algorithms (TALG)*, 3(4):43–es, 2007.

A. Rambaut, E. C. Holmes, Á. O'Toole, V. Hill, J. T. McCrone, C. Ruis, L. Du Plessis, and O. G. Pybus. A dynamic nomenclature proposal for SARS-CoV-2 lineages to assist genomic epidemiology. *Nature microbiology*, 5(11):1403–1407, 2020.

E. Rivas, J. Clements, and S. R. Eddy. A statistical test for conserved RNA structure shows lack of evidence for structure in lncRNAs. *Nat. Methods*, 14(1):45–48, Jan. 2017. doi: 10.1038/nmeth.4066.

F. Tang, J. Chao, Y. Wei, F. Yang, Y. Zhai, L. Xu, and Q. Zou. Halign 3: fast multiple alignment of ultra-large numbers of similar dna/rna sequences. *Molecular Biology and Evolution*, 39(8):msac166, 2022.

S. Triebel, K. Lamkiewicz, N. Ontiveros, B. Sweeney, P. F. Stadler, A. I. Petrov, M. Niepmann, and M. Marz. Comprehensive survey of conserved rna secondary structures in full-genome alignment of hepatitis c virus. *Scientific Reports*, 14(1), July 2024. doi: 10.1038/s41598-024-62897-0.

B. Woolf. The log likelihood ratio test (The G-test). *Ann. Hum. Genet.*, 21(4):397–409, May 1957. doi: 10.1111/j.1469-1809.1972.tb00293.x.
