# Supplementary material for: CREMSA: compressed indexing of (ultra) large multiple sequence alignments
Source: Bioinformatics. 2025 Jul 15;41(Suppl 1):i246–54. doi: 10.1093/bioinformatics/btaf211 (PMC12261481; doi:10.1093/bioinformatics/btaf211)
Supplement: btaf211_Supplementary_Data [file btaf211_supplementary_data.zip › btaf211_Supplementary_Data/Salson.272.sup.1.pdf]

Supplementary material for “CREMSA: Compressed Indexing of (Ultra) Large Multiple Sequence Alignments”

Mikaël Salson Arthur Boddaert Awa Bousso Gueye Laurent Bulteau
Yohan Hernandez--Courbevoie Camille Marchet Nan Pan
Sebastian Will Yann Ponty

March 28, 2025

order	gzip	xz	CoMSA	CREMSA
Random	6	22	42	18
10-discriminative	6	22	42	20
2000-discriminative	7	23	42	21
Length	6	23	42	22
100-random	7	23	42	22
1-random	7	24	42	23
Phylogenetic	7	25	42	24

Table 1: Compression rates on the HIV dataset of 5,381 genomes. Random is a fully random order. The d-discriminative corresponds to the order based on the lexicographic order of d-discriminative subwords shuffled. The d-random corresponds to the order based on the lexicographic order of subwords of length d whose columns were chosen randomly.

order	gzip	xz	CoMSA	CREMSA
Length	7	15	33	15
Random	7	15	33	15
1-discriminative	7	18	33	16
5-random	7	18	33	16
2000-random	10	22	33	21
2000-discriminative	10	22	33	22

Table 2: Compression rates on the MFS dataset of 214,283 sequences. Random is a fully random order. The d-discriminative corresponds to the order based on the lexicographic order of d-discriminative subwords shuffled. The d-random corresponds to the order based on the lexicographic order of subwords of length d whose columns were chosen randomly.

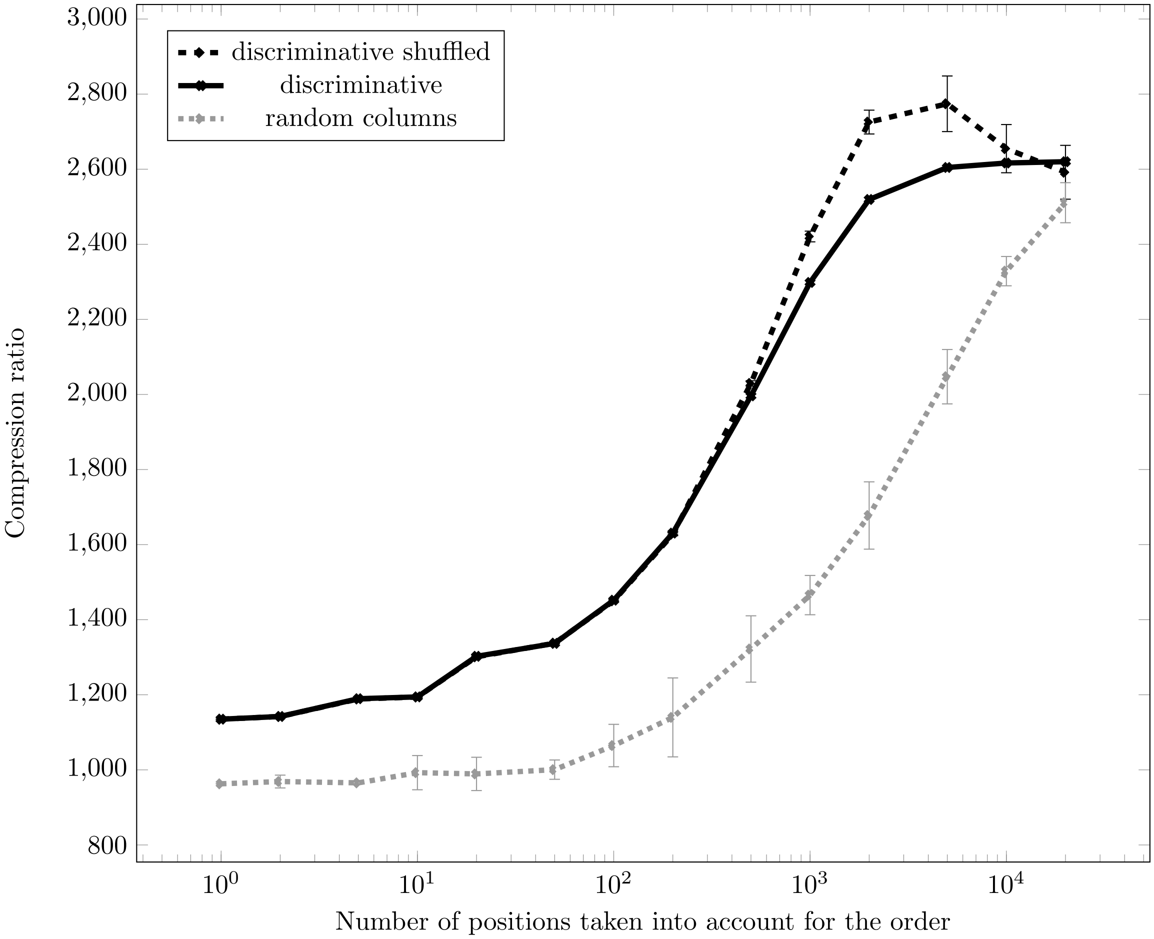

Figure 1: Compression ratio of `CREMSA` on the 1.9M SARS-CoV-2 genomes depending on the size of the subwords user for reordering the sequences. The whiskers on the plot show the standard deviation (computed on 10 trials).

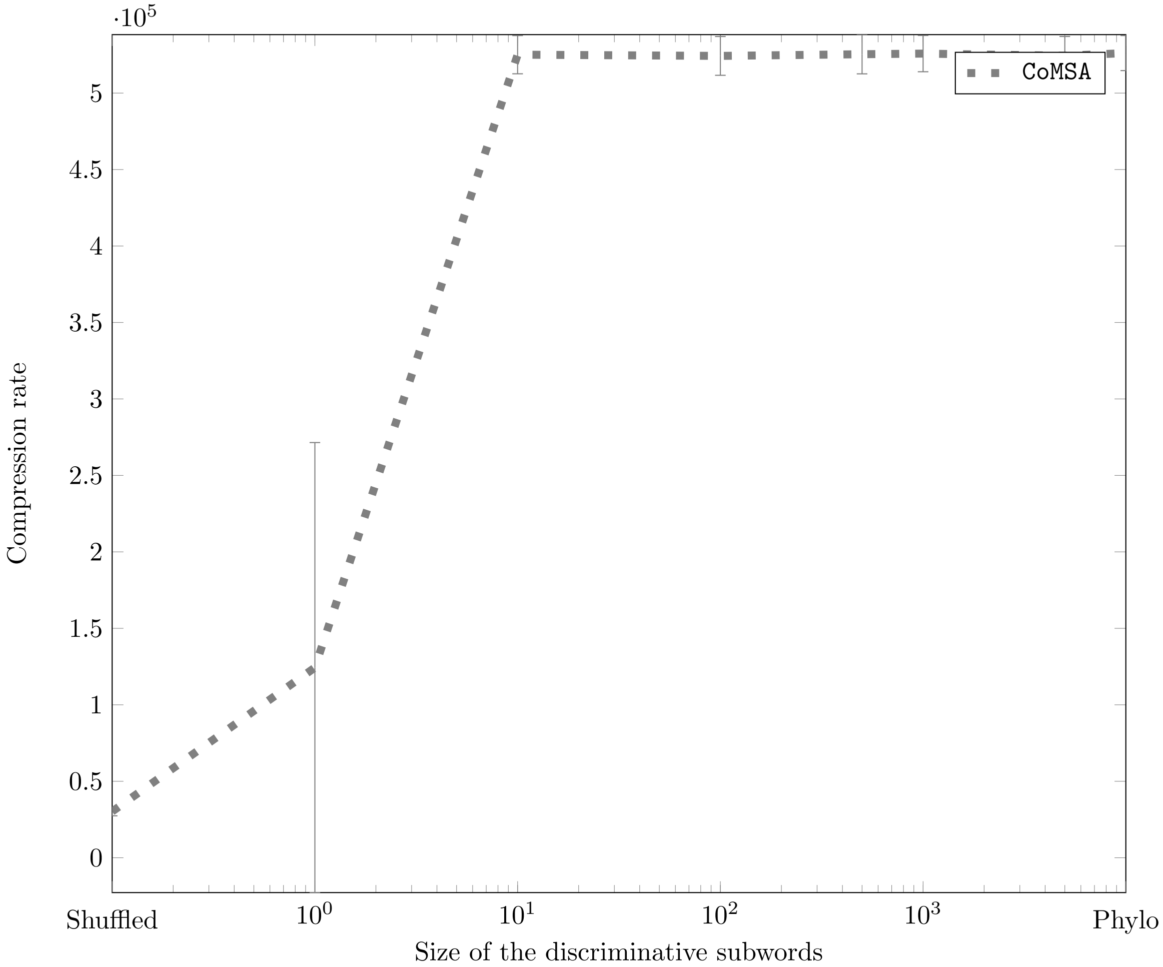

Figure 2: Compression rate of CoMSA on the artificial dataset with .02% mutation rate, depending on the sequence ordering. Shuffled corresponds to the shuffled model in the artificial dataset, while Phylo corresponds to the phylogenetic model. All the values in-between correspond to the shuffled models reordered according to our discriminative shuffled subwords, of different lengths.

data	min	0.01	0.1	1	10	mean	median	90	99	99.9	max
SARS-CoV-2	37.466	50.550	58.490	95.598	99.927	99.717	99.996	100.000	100	100	100.000
HIV	23.304	27.486	32.671	44.750	71.418	92.693	99.610	99.981	100	100	100.000
MFS	10.117	-	12.438	16.717	56.336	91.943	99.978	100.000	100	-	100.000

Table 3: Distribution of the percentage of identitity per column of the multiple sequence alignments of our three datasets. The distribution is shown from selected quantiles, the first 10,000-quantile (0.01), the first 1,000-quantile (0.1), the first percentile (1), the first decile (10), the last decile (90), the last percentile (99), the last 1,000-quantile (99.9) and the last 10,000 quantile (99.99).

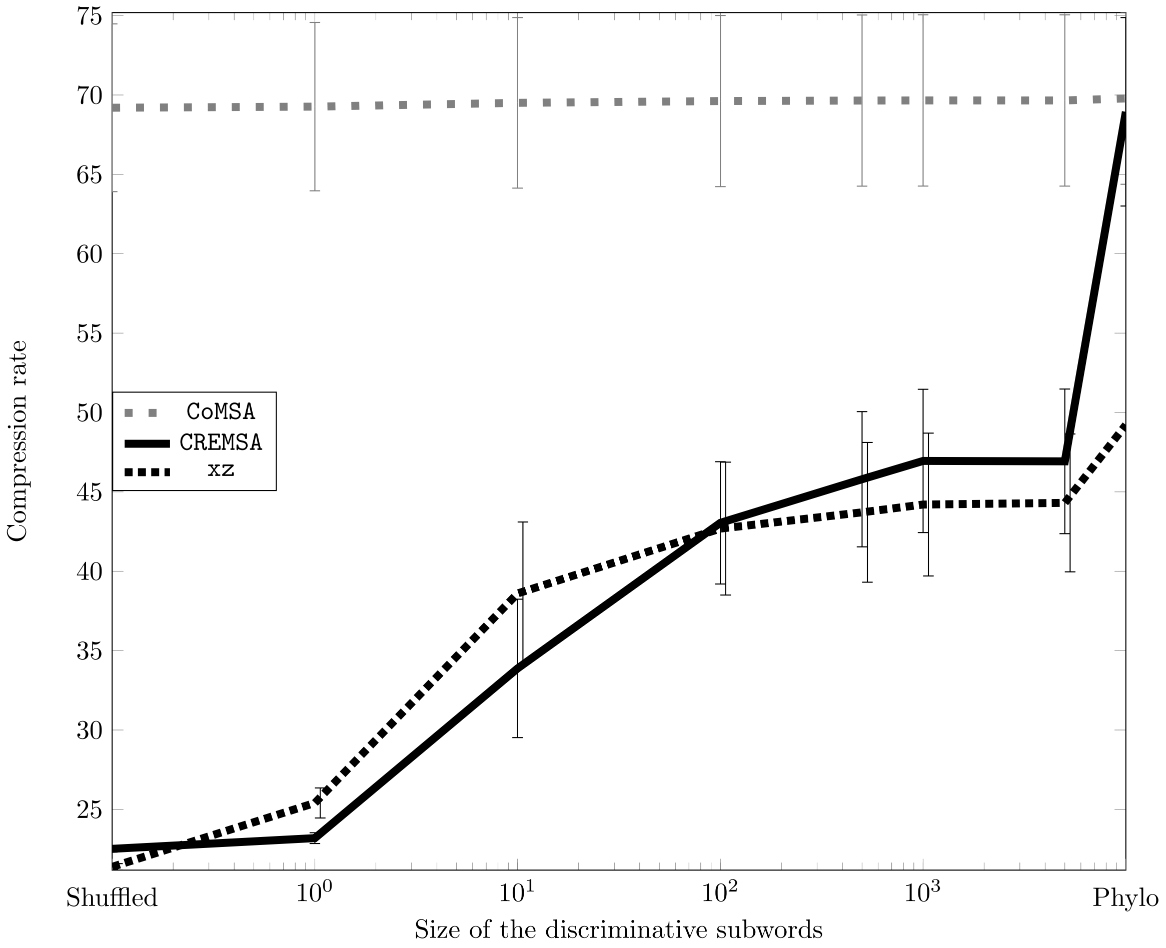

Figure 3: Compression rate of `CoMSA`, `CREMSA` and `xz` on the artificial dataset with 5% mutation rate, depending on the sequence ordering. Shuffled corresponds to the shuffled model in the artificial dataset, while Phylo corresponds to the phylogenetic model. All the values in-between correspond to the shuffled models reordered according to our discriminative shuffled subwords, of different lengths.

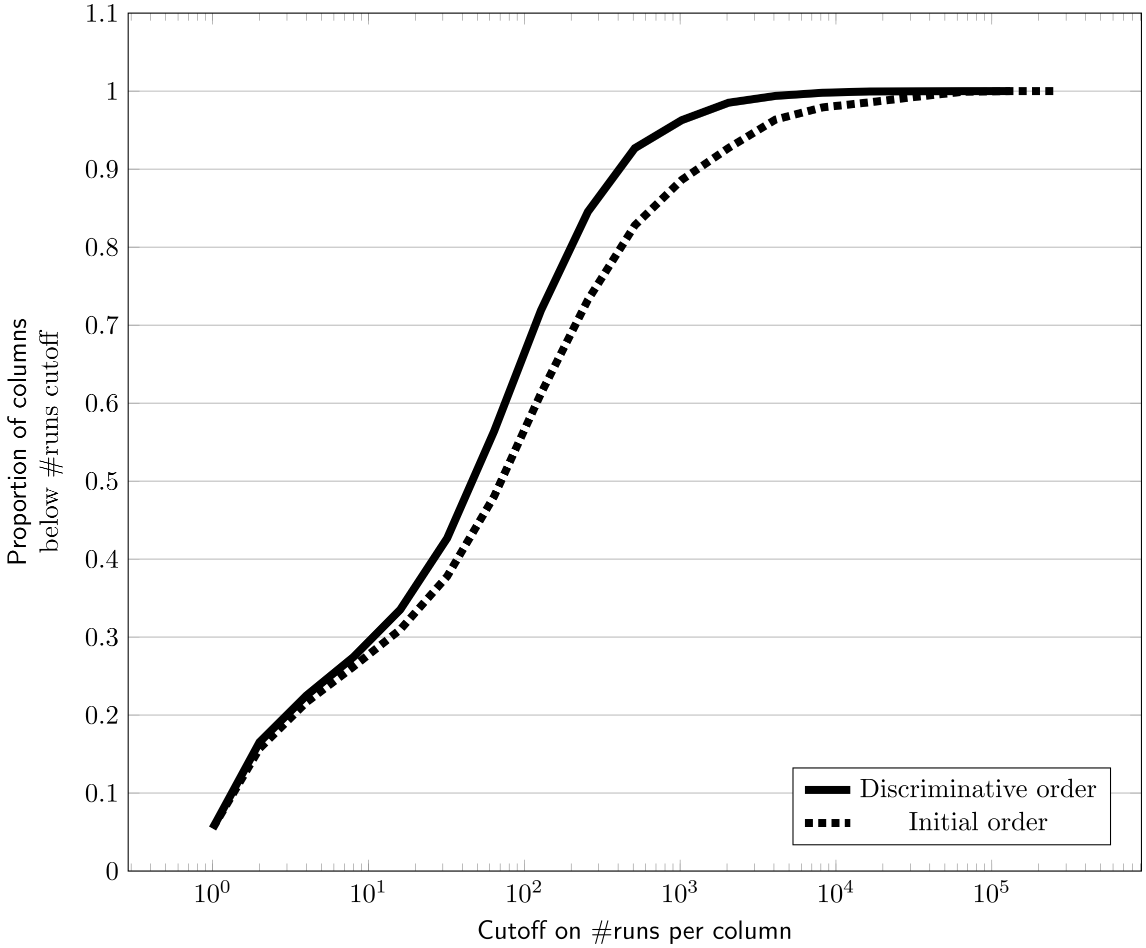

Figure 4: Detailed impact of reordering. Cumulative distribution of the number of runs in each column, as induced by the initial and optimized row ordering. Our optimized *discriminative* order greatly increases the proportion of columns with #runs in the $[10^2, 10^3]$ range, while greatly depleting columns with #runs greater than 5.10^3, resulting in an MSA with improved compressibility.

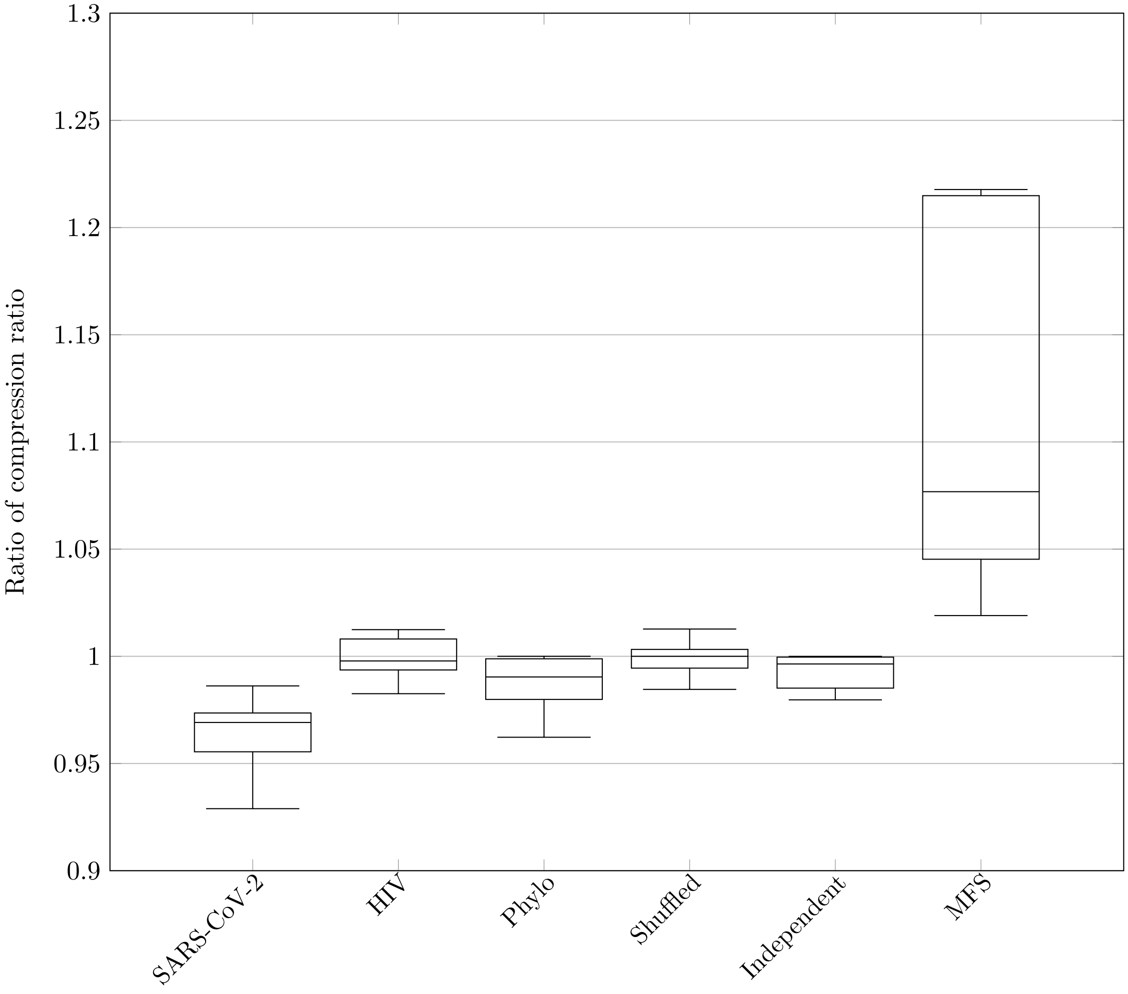

Figure 5: Ratio of the compression ratios between `xz -9` and `xz -6`, for all the datasets considered in the paper. A ratio below 1 means that `xz -6` (the default) compresses better than `xz -9`. All the compressions were run with the option `-T 1`. Phylo, Shuffled Independent refer to the generated sequences using the corresponding model.

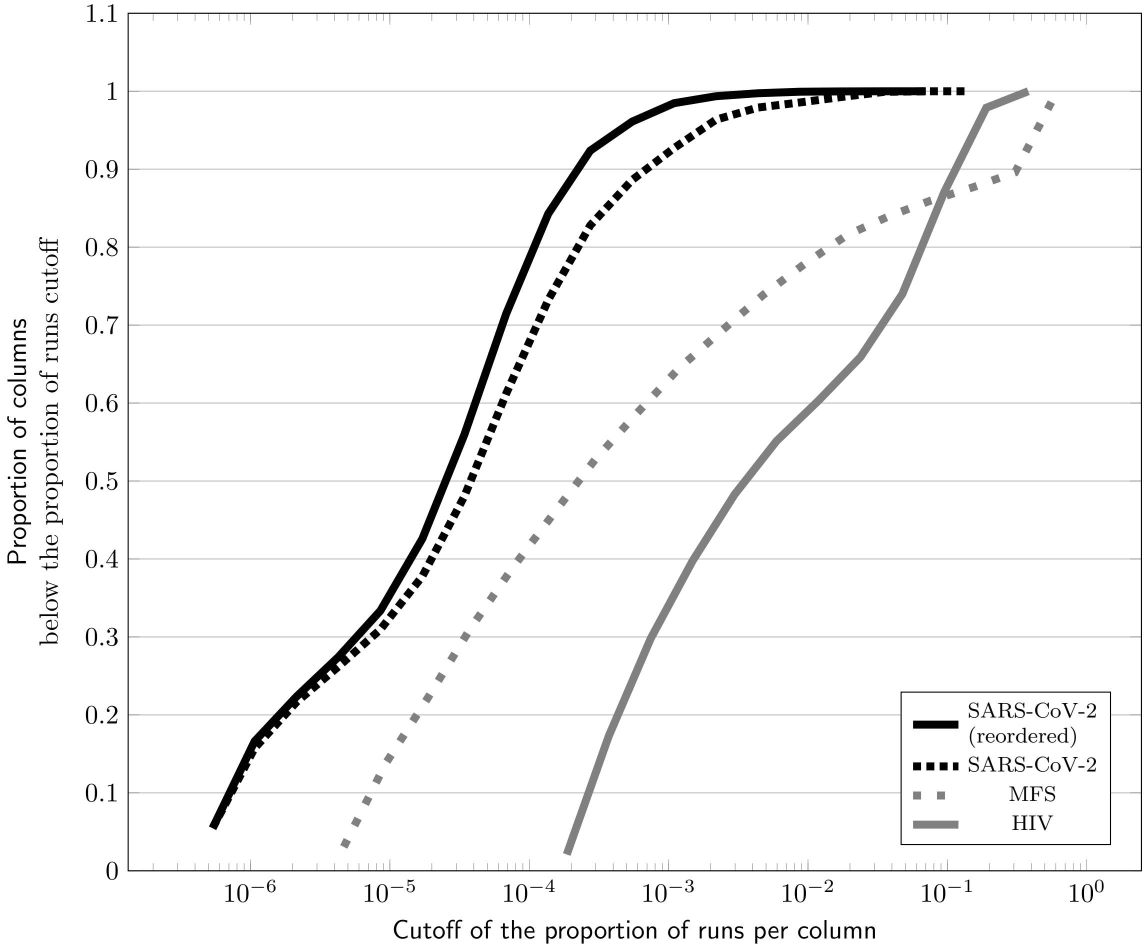

Figure 6: Cumulative distribution of the proportion of runs in each column in the three datasets.
