# Supplementary material for: CREMSA: compressed indexing of (ultra) large multiple sequence alignments
Source: Bioinformatics. 2025 Jul 15;41(Suppl 1):i246–54. doi: 10.1093/bioinformatics/btaf211 (PMC12261481; doi:10.1093/bioinformatics/btaf211)
Supplement: btaf211_Supplementary_Data [file Salson.272.cover.pdf › btaf211_Supplementary_Data/Salson.272.cover.pdf]

Response to reviewers
CREMSA: Compressed indexing of (ultra) large alignments

Mikaël Salson Arthur Boddaert Awa Bousso Gueye Laurent Bulteau
Yohan Hernandez--Courbevoie Camille Marchet Nan Pan
Sebastian Will Yann Ponty

Contents

1 General comments 1

2 Detailed responses 2
Metareview from area chairs 2
Reviewer #1 2
Reviewer #2 3
Reviewer #3 5
Reviewer #4 6
Reviewer #5 7

1 General comments

First of all, the authors would like to express their deepest gratitude for the quality and thoroughness of the reports written by the reviewers, covering a remarkably comprehensive range of aspects of the submitted manuscript. We have individually addressed each of the 51 comments extracted from the first round of review, each to the best of our ability.

We would like to start this response with a mention of the most notable revisions:

- We have further investigated the scope of `CREMSA`, by considering two additional datasets, as well as artificial MSAs generated in realistic and baseline models, showing the applicability of `CREMSA` beyond our initial SARS-CoV 2 dataset;
- We have integrated the online construction algorithm directly within `CREMSA`, which was previously managed by an external tool. This is now the default algorithm and the offline construction algorithm has been discarded.
- We have also integrated the reordering algorithm within `CREMSA`, which performs the reordering directly on the compressed index rather than sorting sequences on disk.
- One of the authors (Thomas Baudeau) expressed the opinion that he did not sufficiently contribute to this work to deserve authorship. Although we disagree and included his name to acknowledge his input during an earlier stage of the project, we have removed him from the list of authors to honor his wish;
- We have fixed numerous typos and unfortunate turns of sentences.

2 Detailed responses

Metareview from area chairs

1. Comment: The reviewers appreciate the method its presentation in your manuscript. At the same time, some concerns are raised and should be addressed before a final decision on acceptance can be reached

   Response: We do appreciate the large number of thorough reviews as well as the opportunity to improve our manuscript through this round of reviews.

2. Comment: Please run your method on at least another data set. Reviewer 5 has some constructive suggestions to this end.

   Response: We have added several datasets in order to answer various remarks from the reviewers:

   (a) a HIV dataset from the Los Alamos National Laboratory;

   (b) a protein dataset consisting of MFS proteins from Pfam, used as a benchmark by earlier work;

   (c) several artificial datasets, to evaluate in more details the effect of diversity and reordering on the compression ratio.

   Our results show that `CREMSA` can indeed be executed in this setting and works reasonably well. Predictably, the compression ratio predictably suffers in MSAs resulting from an aggressive sub-sampling (*e.g.* 20x for the HIV/MFS, both of which have 92% avg identity). Yet, `CREMSA` remains competitive with text-based compressors (`xz`) and offers accelerated downstream capabilities.

   We have also integrated a phylogenetic-tree based order as suggested by Reviewer 5 in our updated Table 2. The order we introduce still provides better compression rate than this new phylogenetic ordering.

3. Comment: Provide more context on how your method relates to existing approaches, such as the PBWT and others.

   Response: We now discuss this in the paper, in the Discussion section. We believe that p-BWT or r-index or any BWT-based index cannot easily count efficiently all the possible tuples in any tuple of columns. Actually, what BWT-based indexes are able to do is to efficiently search for a given pattern. This is not our application case: we do not want to search a given pattern in the MSA but we want to enumerate all the tuples of letters in any tuple of columns. In this application, the BWT cannot compute efficiently those queries of interest, it would require to uncompress the tuples of columns in order to enumerate all the tuples of letters.

4. Comment: While these are the most important points, please aim to also address other comments made by reviewers, especially when they ask for clarifications on details of the method and evaluation.

   Response: We have tried to address each and every reviewer's comments in a way that will hopefully be found satisfactory by the Area Chairs.

Reviewer #1

5. Comment: The paper is reasonably well written and the experiments are well designed and described. The experimental results are good.

   Response: We appreciate the encouraging words.

Reviewer #2

6. Comment: Major 1. This paper uses only a SARS-CoV-2 dataset of around 1.9M sequences for both the analysis of their tool's compression abilities and to demonstrate the potential for novel biological analysis. Despite this being a large dataset, it has a lot of redundancy on the column level and it has relatively short sequences. It is not clear how this tool will perform if the genomes in the dataset are not as conserved or are longer. Also, it is not clear whether the biological analysis they have shown to be feasible with their tool will also be feasible for other datasets. It would strengthen the paper to include other datasets in the evaluation.

   Response: Clearly, the performances of the method will smoothly degrade with the number of runs, itself being strongly impacted by a decreasing sequence identity.

7. Comment: Major 2. To prepare the dataset, this paper gets rid of sequences that have at least two consecutive Ns (except at the end). In reality, however, a user may be working with sequences with some Ns, and it would be good to know how the tool performs in those scenarios.

   Response: Firstly, we must point out that do not rule out Ns altogether, we simply use the presence of consecutive N as a proxy for low-quality sequences. Namely, the sequences we retained do have Ns and all other non-ACGT IUPAC characters: Among the 1.9M aligned genomes (totaling 65 Gbp), we have 87,952,143 such characters in our initial dataset.

   Moreover, while it is clear that sequencing errors will lead to more diverse sequences, which would be harder to compress, this preprocessing purpose is to remove sequences that would add too much noise in the multiple sequence alignment. We added in the Material and Methods an information on the MSA obtained on a subsample of unfiltered genomes: on 449k genomes, the MSA has 230,491 columns.

8. Comment: Major 3. It is not clear whether using d-discriminative subwords heuristic is a two step process. That is, does a user need to create a CREMSA index first, find these subwords, sort the genomes accordingly, and create a CREMSA index again? What would be the runtime or memory usage for doing all these steps? Also, I could not find how much time it takes to find all the d-discriminative words.

   Response: This has now been specified at the end of the section "Improving compressibility through sequence reordering". This is also clearer since the process is now integrated within CREMSA. Table 1 now integrates the time and memory of the whole process.

9. Comment: Major 4. It seems to me that the idea of the d-discriminative subwords heuristic is underdeveloped. The subwords are created according to the ordering of the lowest percentage of identity. The intuition for doing this was not completely clear to me and in fact, the analysis showed that a different ordering does not impact the performance. For example, if I have three 4-subwords, I can order each of them in different ways and will it still not impact performance? It would be helpful if you clarify why this might be the case. Also, I think it would improve clarity if there are some examples/figures explaining the effectiveness of the heuristic.

   Response: Indeed, this observation is astute and fascinating. We investigated the problem from a theoretical perspective, and found minimal counterexamples where the order already matters. For instance, the following pair of orders for the same set of sequences, leads to different #runs:

Order	2 $\leftarrow$ 1		Order	1 $\rightarrow$ 2	
	A	A		A	A
	B	A		A	B
	C	A		B	A
	A	B		B	B
	B	B		C	A
	C	B		C	B
8 #Runs:	6	2	9 #Runs	3	6

The observed stability of the number of runs upon reordering may be indicative of a concentrated distribution, over all permutations of genomes, of the #runs, or could be a specific feature of sequences resulting from an evolutionary process. Regardless of the final conclusion, a satisfactory study of this phenomenon would require a dedicated study, whose scope would clearly exceed the scope of the current manuscript.

10. Comment: Major 5. It is not clear how a user should choose the d value for this heuristic approach or how the performance varies with d size.

    Response: We added Supplementary Figure 1, which showcases the effect of d on the compression ratio for the 1.9M SARS-CoV-2 genomes dataset. On the artificial datasets, we also added Figure 3 as well as Supplementary Figures 2 and 3. Finally, we added a discussion on choice of a value for d in the Results and Discussion sections.

11. Comment: Major 6. The running time and memory usage in Table 1 do not include the performance of the python script used for reordering the sequences, thus making it a slightly unfair comparison. This is because, from Table 2, the initial order gives CREMSA a compression ratio of 965 which is smaller than both xz and CoMSA. However, only after they reordered the sequence according to the heuristic, they showcased improvement in compression ratio than xz. Thus, without including the memory and time usage for the heuristic, this comparison becomes unfair.

    Response: The process is now integrated in CREMSA, and Table 1 now shows the time both for the index construction alone and with the reordering.

12. Comment: Major 7. In Table 1, I could not understand whether the memory usage or the compression ratio of CREMSA is from the zipped version or the unzipped version.

    Response: Both are in comparison to the unzipped version. We believe to be fair since any alternative method would require decompressing the alignment before enacting its own compression.

13. Comment: Major 8. The title is too general. At the least, it should refer to multiple sequence alignment specifically, rather than to alignment generally.

    Response: We added "Multiple Sequence" in the title.

14. Comment: Minor 1. I think it would improve clarity if a theorem is used to prove that finding an optimal ordering is an NP-complete problem instead of proving this in the middle of the text.

    Response: Given the space limitations, and the relative simplicity of the main argument underlying our reduction, we must unfortunately decline and keep this proof inlined.

15. Comment: Minor 2. "The current MSA representation remains the similar than the main construction algorithm, where compressed bit vectors are associated to strings for columns representation." - this line is difficult to understand.

    Response: Indeed, our wording was probably confusing. This part has been fully rewritten as now the Online construction has been integrated in CREMSA and the offline construction has been discarded.

16. Comment: Minor 3. Typo: "For a length-n bit vector B, $rank_1(B, i)$ if the number of..." - it should be "is" instead of "if".

    Response: Indeed.

17. Comment: Minor 4. Typo: Multiple places have used "disciminative subwords" instead of "discriminative subwords".

    Response: Indeed.

18. Comment: Minor 5. In Figure 2, I think the x-axis should be "Chunk Size" instead of "Number of Chunks".

    Response: The figure has now been changed (now Fig 4), to depict the time/space consumption of the online construction integrated to CREMSA.

Reviewer #3

19. Comment: The authors refer to CREMSA as an ”index structure”, even a ”self index”. But what is actually indexed here? What we have is the compressed representation of an MSA, but no indexing. Granted, counting and entropy queries across different columns are supported, but to me, ”index” implies some sort of lookup query.

    Response: In CREMSA, the columns are indexed. They are indexed using bit vectors with rank/select capabilities. The data structures enabling efficient rank/select operations are called indexes themselves (see 10.1007/978-3-642-27848-8_332-2). Our index supports lookup query as we can recover any position from the original MSA in constant time. On top of that, our index support counting queries to count the occurrences of all the tuples of symbols in any tuple of columns.

20. Comment: How is the compression actually implemented? There is a ”compressed bit vector”, but how exactly? Which data structure for select is used? Is access really constant-time. Practical implementations exist that have worse theoretical guarantees but are fast in practice. I suppose these questions are finally answered in the Results section, but this belongs to the Methods. Also, a theoretical analysis is given in Methods that differs from the practical implementation.

    Response: Indeed, the theoretical methods and the practical ones can differ. From the theoretical standpoint, we have constant-time rank/select operations on efficiently compressed bit vectors. In practice, other considerations matter, beyond the theoretical complexities: such as the libraries available for rank/select operations, their practical efficiency and so on. That is why there is a difference on the complexities between the Methods and the Results. But the complexities of the structure we use were already clearly stated. We added, at the start of the Results section, what are the compression methods used by the SD vector and the RRR vectors.

21. Comment: The hard part, actually obtaining the multiple sequence alignment, is not really factored into the process. Typically this would also have to be done in a divide and conquer fashion, or on a much larger machine, and this would have a direct influence on how and when to compress.

    Response: We agree with the reviewer that computing the multiple sequence alignment is difficult on a large amount of data, however this is beyond the scope of this paper.

22. Comment: Important: The comparison should really consider meaningful baselines, such as bzip2 on the rows or columns of the MSA. It is also not clear whether xz and gzip were applied to the row-wise or column-wise data. It is also not clear if the compression ratio starts from bytes (8 bit/character) or already bit-encoded data (2.something bits/character).

    Response: We understand that it would be interesting to know how good the columns can be compressed using baseline compressors. However retrieving the MSA column-wise is not trivial, so to compress them efficiently would not be straightforward. Moreover, we believe that we should compare to the readily available methods. Finally, we did not include it in the paper (for a matter of space) but the columns do not compress better with `xz` compared to the rows on the SARS-CoV-2 dataset, while of course `gzip` does (but still performing poorly in comparison to `xz`).

23. Comment: What about building an r-index? Is it feasible? If it is built column-wise one maybe has similarly efficient k-tuple counting queries.

    Response: As indicated in the answer to the metareview above, we now address this issue in the Discussion.

    -

24. Comment: Any relation to positional BWT (pBWT), since one has a fixed rectangular scheme?

    Response: Ibid.

    -

25. Comment: Do the tools run single-threaded or somehow in parallel?

    Response: In the results, we mentioned that the experiments were performed on a single thread. However we added the fact that CoMSA cannot be restricted to a single thread.

26. Comment: The genome is only 30Kbp, but there are almost 35K columns. This can be explained of course by diversity, but it raises the general question whether the MSA is in a biological sense "correct" and whether MSAs are even the right concept to investigate such a large number of genomes. Maybe this could at least be commented. For example, co-evolution between genomic parts could also be considered by co-changed k-mers, etc.

    Response: Horizontal expansion, in this specific case, more probably reflects accumulated errors, both on the sequencing side and during the heuristic construction of the MSA, rather than an intrinsic difficulty of MSAs to represent viral evolution.

27. Comment: Minor. Abstract: genomesin -¿ genomes in

    Response: Indeed

28. Comment: "Improving compressibility through sequence reordering": percentage of identity → fraction of identity, what you define is not a percentage. Also, please do not use \frac in running text, but a simple division (/).

    Response: Indeed, we changed percentage of identity into fraction of identity.

29. Comment: "Entropy and covariation metrics for RNA comparative analysis": Here, the column index is i, but it was j before.

    Response: Indeed

30. Comment: Fig.2: Axes (here: time) should always start at 0, not 25 or 30?.

    Response: We respectfully disagree with the reviewer. However for a matter of space Fig. 2 is now a table (Table 3) and the only plot with time on the y-axis (Fig. 5) has a logarithmic scale where starting at 0 is not feasible.

Reviewer #4

31. Comment: The authors write that the time complexity for counting nucleotide pairs in two columns is $\Theta(max(r_{j_1}, r_{j_2}))$. Shouldn't this be something like $\min(s, r_{j_1} + r_{j_2})$? For instance the columns (AACCGGT) and (ACCGGTT) have number of runs $r_{j_1} = 4$ and $r_{j_2} = 4$, but the number of pairs is 7 (AA, AC, CC, CG, GG, GT, TT).

    Response: The suggested complexity is asymptotically equivalent to the one provided in our article. Indeed, one has

$$\max(r_{j_1}, r_{j_2}) < r_{j_1} + r_{j_2} < 2 \times \max(r_{j_1}, r_{j_2})$$

    which implies that $r_{j_1} + r_{j_2} \in \Theta(max(r_{j_1}, r_{j_2}))$.

32. Comment: The algorithm is designed for very similar sequences. How similar is similar enough and from what distance on the compression of CREMSA is no longer effective?

    Response: We simulated increasingly divergent MSAs in order to answer this question. As shown in Fig. 2, the compression rate obviously decreases for all the methods when the mutation rate increases. However `CREMSA` still performs similarly to `xz` for the higher mutation rate (5%). Our experiments on the Pfam dataset also shows that CREMSA can cope with much more dissimilarity (as expected with protein sequences), see Supplementary Table 2.

33. Comment: In the conclusion the authors write: "We also compute an index on 1,870,492 SARS-CoV-2 genomes aligned with Halign3 (65GB of data) in an index compressed to 22MB on disk and taking 81MB of RAM." However, in Table 1 the Memory for CREMSA is given as 326 GB. So which one is it? Or is Table 1 peak memory during compression?

Response: The difference between both, as suggested by the reviewer, is that Table 1 shows peak memory during the construction while the space shown in the Conclusion corresponds to the exact space required by the final in RAM (ie. the amount of RAM used when querying it). We have clarified the conclusion and the caption of Figure 3 to make clear what was measured.

34. Comment: Minor. The authors write: "More formally, we define B_j of length n ..." –¿ B_j should be of length s, as n is the number of columns.

    Response: Indeed, thanks for spotting the typo. This has been fixed.

35. Comment: Minor. The text says: "The current MSA representation remains the similar than the main construction algorithm, ..." –¿ I don't understand this sentence

    Response: This part has now been fully rewritten since the index is now only constructed online.

36. Comment: Minor. The text says: "Under the best order, in CREMSA 1,473 bit vectors are stored using a RRR bit vector, this number increases to 4,214 for the initial order." –¿ I had to read this 5 times before I understood. The authors describe the fraction of bit vectors that is stored as RRR and not as SD. Please make this more clear.

    Response: We changed the sentence to make it clearer, hopefully.

37. Comment: Typo. -Abstract: genomesin –¿ genomes in

    Response: Indeed

38. Comment: Typo. "disciminative subwords" –¿ discriminative subwords

    Response: Indeed

39. Comment: Typo. "Let G_1, ..., G_s the genomes" –¿ "be" missing in front of "the"

    Response: Indeed

40. Comment: Typo. "is less time-efficient that the one used" –¿ is less time-efficient than the one used

    Response: This part has now been reformulated

41. Comment: Typo. Fig 6 caption: "which exceeding" –¿ which exceed

    Response: Indeed

Reviewer #5

42. Comment: 1) The title refers generally to large alignments, but the entire paper is about SARS-CoV-2 only, and generalizability to other types of genomes is unclear (and even to other viruses, and even to less clean SARS-CoV-2 datasets). Either the benchmarking should include other viruses (note that many datasets for Influenza, HIV, Ebola, etc. are readily available – on GISAID, etc.) and then a reference to "viruses / viral" should appear in the title, or the title should talk specifically about SARS-CoV-2. (If the method's ambition is to go beyond viruses, e.g., to core genome alignments of a bacterial species, it should be benchmarked on the corresponding data as well.)

    Response: We believe that `CREMSA` can be of interest beyond the study of viruses. This is why we have now included a Pfam dataset on a protein family.

43. Comment: 2) The term "phylogenetic order" is used in a confusing way – what the authors claim by the "phylogenetic order" is in fact genomes sorted according to pangoline lineages, so only by clusters. So this should be called a "lineage order", instead. Moreover, the concept of pangolin lineages should be at least briefly explained, and the impact of the choice of a specific lineage system on the performance assessed as well. These comparisons may be non-trivial and need to be done properly.

Response: We clarified this and we integrated a real phylogenetic order, as suggested in the following comment. Our results now present both. We show that, on the SARS-CoV-2 dataset, the best order for CREMSA is still the one we introduce in our paper, based on discriminative subwords.

44. Comment: 3) A "real phylogenetic order" is currently missing in Tab 2 and should definitely be included in this benchmark. This should correspond genome sorted according to the phylogenetic tree – e.g., the leave order within the post-order enumeration. In SARS-CoV-2 research, phylogenetic trees are almost always available and provided alongside assemblies / MSAs – especially for major datasets such as GISAID or the [Hunt et al, 2024]. (In the case of the used dataset, the tree is provided on taxonium.org and should be extractable by the matUtils package. Therefore, such a comparison should be trivial.)

    Response: We are grateful to the reviewer for the suggestion and for the detailed information to retrieve the phylogeny for our dataset. We have now included an ordering based on the phylogenetic tree for the SARS-CoV-2 dataset as well as the additional HIV dataset. We now show in the paper that our order on discriminative order is still better for CREMSA on the SARS-CoV-2 dataset than the order based on the phylogenetic tree.

45. Comment: 4) The xz results in Table 1 look intriguing and are not sufficiently explained / highlighted. We managed to reproduce the surprising phenomenon that xz with the default parameters provided better compression than xz -9. Given the non-intuitivity of this, the results should be properly documented and provided eg in the supplement as this might be useful for the community. It should be also be documented how exactly the compression was done – using -T1?

    Response: We thank the reviewer for reproducing our observation. We have run `xz` with both options on all our datasets and show these results in Supplementary Figure 5.

46. Comment: 5) The workflow in Snakefile does not seem to be reproducible. We tried to run it on our mac computers, but ran into conda issues. It seems to requires gxx-linux64 (a dependency in build_env.yml), but this needs to be documented clearly, as well as how to run software and what hardware/software is necessary. When we tried, in our other attempts, to make it work on our Linux cluster, it also failed with errors such as

    ```
    Error:  /usr/include/c++/4.8.2/bits/c++0x_warning.h:32:2:  error:
    #error This file requires compiler and library support for the ISO C++
    2011 standard.  This support is currently experimental, and must be
    enabled with the -std=c++11 or -std=gnu++11 compiler options.
    ```

    Response: We thank the reviewer for pinpointing those issues. We relaxed the dependency, now on `gxx` rather than `gxx-linux64`. Regarding the compilation issue, as indicated in the README on CREMSA repository, the compilation should be achieved with a C++-17 compiler. The one used here seemed to be g++ 4.8.2, while the first g++ version to fully support c++-17 was g++-8.

47. Comment: Minor. 1) In the abstract of the paper, there is a typo in the first phrase: "...genomesin order to..." → should be "...genomes in order to..."

    Response: Indeed

48. Comment: Minor. 2) Installation issue on macOS (M4 chip): The provided offline version cannot be installed directly on macOS with the M4 chip. To install CREMSA, the Makefile must be modified to specify the C++ version. This issue can be resolved by adding "-std=c++17".

    Response: The Makefile has been modified accordingly. At the same time, we emphasize the fact that CREMSA can also be run with Docker, without the need for compiling it.

49. Comment: Minor. 3) Unclear phrase on page 3: On the third page, second column, second paragraph, the phrase "The current MSA remains the similar than the main construction algorithm ..." is unclear. The intended meaning should be clarified.

Response: This part has now been reformulated to reflect the fact that `CREMSA` is now only built online.

50. Comment: Minor. 4) It is unclear if there are any assumptions on the alphabet. What happens if some of the assemblies contain N?

    Response: For a given number of runs, the performance of our method is alphabet-agnostic, beyond a $\lceil \log |\Sigma| \rceil$ linear factor being induced by unbounded alphabet. Of course, for very diverse sequence datasets, larger alphabets will typically imply shorter/more numerous runs, hindering compressibility. In particular, `N` characters are supported without noticeable cost.

51. Comment: Minor. 5) Similar strategies have been studied in older genomic compression papers. It would be nice to put it better into the context of papers such as 10.1093/bioinformatics/btp117.

    Response: Indeed, column-wise compression have been utilized in various bioinformatics methods, and the reference is relevant. We now indicate that our method is related to the table compression methods.
